# Thomas on Extra-Uterine Pregnancy

**Published:** 1883-03

**Authors:** 


THOMAS ON EXTRA-UTERINE PREGNANCY.—In a paper entitled "Notes of Twenty-one Cases of Extra-Uterine Pregnancy," read at the last meeting of the American Gynecological Society, the author, Dr. T. Gaillard Thomas gave the following rules with reference to treatment: 1st. If an ectopic tumor were discovered and its nature pretty well settled before the end of the fourth month of gestation, he would destroy the vitality of the child by electricity in preference to all other methods which had been proposed. It had these great advantages: If an error of diagnosis had been made, this remedy would do no harm; if the diagonosis was correct experience proved it to be sufficient in its effects. It was almost painless, and caused none of the nervous disturbances created by a cutting operation, and it required no surgical skill in its use. 2d. Should the fourth month of gestation be passed and surgical interference be called for, he thought that laparotomy or, if the tumor were low down in the pelvis, elytrotomy should be preferred to the use of electricity, which left a large fœtal body to undergo absorption inside the body of the mother. 3d. Should the pregnancy be abdominal, the practitioner might watchfully await the full term of gestation, and deliver then, by laparotomy or by elytrotomy, combined with the forceps of version. 4th. Should full term be passed, and the fœtus be dead, the practitioner should wait and watch, if possible, until nature demonstrated the outlet by which she desired the extrusion to be effected; then she should be aided. If, on the other hand, bad symptoms under these circumstances at any time showed themselves, laparotomy, under strict antiseptic precautions, should be promptly resorted to. 5th. Should rupture of the fœtal nidus have occurred before a diagonosis had been fully made, the practitioner should wait and see whether nature was powerful enough to overcome the shock and control hæmorrhage, then, further, if the patient was going to escape the dangers of peritonitis and septicæmia. If these favorable results did not occur, if hemmorhage was about to destroy the patient immediately, or if septicæmia attacked her, laparotomy, followed by antiseptic clensing should be promptly adopted.

